# Nimodipine but Not Nifedipine Promotes Expression of Fatty Acid 2-Hydroxylase in a Surgical Stress Model Based on Neuro2a Cells

**DOI:** 10.3390/ijms18050964

**Published:** 2017-05-03

**Authors:** Eva Herzfeld, Lea Speh, Christian Strauss, Christian Scheller

**Affiliations:** Department of Neurosurgery, Martin-Luther University of Halle-Wittenberg, Ernst-Grube-Str. 40, 06120 Halle (Saale), Germany; lea.speh@gmx.de (L.S.); christian.strauss@uk-halle.de (C.St.); christian.scheller@medizin.uni-halle.de (C.Sch.)

**Keywords:** nimodipine, nifedipine, neuroprotection, Neuro2a, stress, FA2H, myelin

## Abstract

Nimodipine is well characterized for the management of aneurysmal subarachnoid hemorrhage and has been shown to promote a better outcome and less delayed ischemic neurological deficits. Animal and clinical trials show neuroprotective efficacy following nerve injuries. We showed a neuroprotective effect on Neuro2a cells. Subsequent microarray analysis revealed—among others—fatty acid 2-hydroxylase (FA2H) upregulated by nimodipine in vitro, which is a component of myelin synthesis. Differentiated Neuro2a cells were analyzed for nimodipine-mediated survival considering stress treatment in comparison to nifedipine-treatment. Cell survival was determined by measurement of LDH activity in the culture medium. Nimodipine decreased surgery-like stress-induced cell death of differentiated Neuro2a cells. Neuro2a cell culture was analyzed for changes in FA2H expression induced by nimodipine or nifedipine in surgery-like stress conditions. We analyzed expression levels of FA2H mRNA and protein by qPCR using *fa2h* specific primers or a FA2H-specific antibody in nimodipine or nifedipine non- and pre-treated Neuro2a cell culture, respectively. Nimodipine but not nifedipine increases FA2H protein levels and also significantly increases mRNA levels of FA2H in both undifferentiated and differentiated Neuro2a cells. Our findings indicate that higher expression of FA2H induced by nimodipine may cause higher survival of Neuro2a cells stressed with surgery-like stressors.

## 1. Introduction

Nimodipine as well as nifedipine are 1,4-dihydropyridine L-type calcium channel antagonists. Nifedipine is used in the treatment of hypertension [1], the vasospastic form of angina pectoris (Prinzmetal’s angina) [2], Raynaud’s disease [3], and premature labor [4]. Nimodipine is recommended for the management of aneurysmal subarachnoid hemorrhage (aSAH) and has a proven effect in reducing poor outcome and delayed ischemic neurological deficits (DIND) following aSAH [5,6]. In skull base, laryngeal and maxillofacial surgery in animal experiments [7,8,9,10], and clinical series it shows a beneficial effect [11,12,13,14,15,16]. These results were linked to neuroprotection described by Nuglisch et al. [17]. An off-label use of oral nimodipine improved the regeneration of peripheral facial nerve paresis after maxillofacial surgery [14]. Also nifedipine shows neuroprotective activity at least on dopaminergic substantia nigra neurons in rats [18]. Neuroprotection by nimodipine but not by nifedipine was shown in nerve growth factor (NGF)-differentiated PC12 neuronal cells [19]. Recently we showed that the survival of Neuro2a cells was significantly higher when cells were pre-treated with nimodipine prior to oxidative, mechanical and heat-induced stress [20]. These represent stressors, which may occur during surgery as described before [20]. Besides mechanical stress, nerve tissue may be affected intraoperatively by an increase in temperature caused by the microscope and the bipolar coagulation or by dryness and ionic shifts. Based on this study we analyzed the cell survival of differentiated Neuro2a cells and the protective effect of nifedipine on Neuro2a cells. Gene expression pattern of nimodipine-treated and non-treated cells by microarray were analyzed to get insight into molecular processes linked to the neuroprotective effects of nimodipine. One of the differentially regulated genes was analyzed in more detail. Fatty acid 2-hydroxylase (FA2H) is crucial for de novo synthesis of 2-hydroxy fatty acids, which are incorporated in 2-hydroxy galactolipids. These are thought to play important roles in lipid-lipid and lipid-protein interactions in myelin [21,22,23,24,25]. In the present study, we analyzed FA2H mRNA levels regulation in dependence on nimodipine but not on nifedipine.

## 2. Results

We found, according to recent data in undifferentiated Neuro2a cells, that also differentiated Neuro2a cells are protected from mechanical, heat and oxidative stress by nimodipine, or at least to a lower extent by nifedipine. We analyzed the transcription of FA2H in dependence on nimodipine or nifedipine treatment, respectively. We observed that nimodipine but not nifedipine significantly increases the expression of FA2H mRNA in Neuro2a cells up to 10× (undifferentiated cells, *p* < 0.0001) or 25× (differentiated cells, *p* < 0.0001) depending on general fitness of the cells compared to reference cells. In contrast to this, the FA2H mRNA expression is decreased or increased by the stressors themselves. We also detected higher FA2H protein levels in nimodipine but not nifedipine pre-treated Neuro2a cells.

### 2.1. Survival of Nimodipine-Treated Differentiated Neuro2a Cells

Cell death was determined by measuring lactate dehydrogenase (LDH) activity in cell culture supernatant.

**Figure 1:**
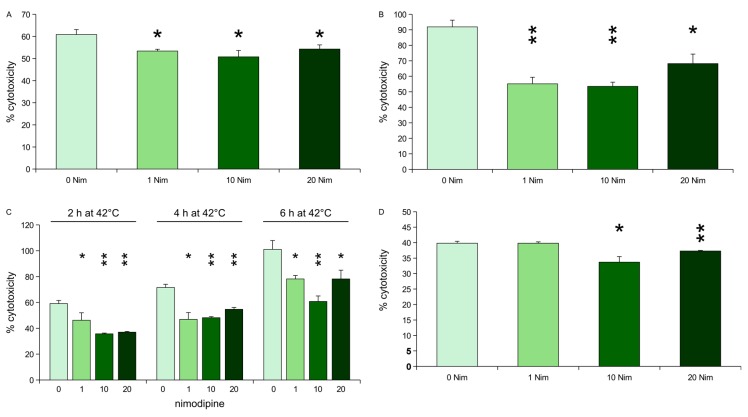
Lactate dehydrogenase (LDH) activity measurement after stress on differentiated, nimodipine pre-treated Neuro2a cells. Values are given as the mean ± SD (error bars) of one representative out of at least three biologically independent experiments. Nim = nimodipine (in µM); single asterisk = *p* < 0.05 compared to non-treated cells; double asterisks = *p* < 0.005 compared to non-treated cells. (**A**) Oxidative stress (EtOH, 2%); (**B**) osmotic stress (NaCl, 150 mM); (**C**) heat stress (4 or 6 h at 42 °C, respectively); (**D**) mechanical stress.

Osmotic stress was induced by treating the nimodipine pre-treated cells and untreated cells with 150 mM NaCl. All tested nimodipine concentrations led to a reduced cytotoxicity. 1, 10 or 20 μM nimodipine reduces the cytotoxicity of NaCl from 91% (untreated cells) to 55 (*p* = 0.0005), 53 (*p* = 0.0002) and 68% (*p* = 0.0055), respectively (Figure 1A).

1 µM nimodipine did not reduce cytotoxicity of 1.8% EtOH (40%, *p* = 1). 10 or 20 μM nimodipine reduced the cytotoxicity of EtOH slightly but significantly to 33% (*p* = 0.0052) and 37% (*p* = 0.0029), respectively (Figure 1B).

Mechanical stress was induced by adding two steel beads (2 mm) to each well of a 24 well-plate of pre-treated or untreated cells, respectively, and shaking the plate at 500 rpm for 30 s. Nimodipine reduced the cytotoxicity from 61% (untreated cells) to 53% (1 μM nimodipine), 51% (10 μM nimodipine) and 54% (20 μM nimodipine, *p* < 0.005 each) (Figure 1C).

Heat stress was induced by transferring the nimodipine pre-treated cells and the control cells to 42 °C for 2, 4 or 6 h, respectively. After heat incubation, cells were returned to 37 °C. All tested concentrations of nimodipine led to a reduction of cytotoxicity induced by heat. When incubated at 42 °C for 2 h, nimodipine reduces cytotoxicity from 60% to 46%, 36%, and 37% (*p* < 0.005 each), concerning 4 h heat incubation from 71% to 47%, 48%, and 54% (*p* < 0.005 each), concerning 6 h heat incubation from 100% to 78%, 61%, and 78% (*p* < 0.005 each) for 1, 10 and 20 µM nimodipine, respectively (Figure 1D).

### 2.2. Survival of Nifedipine-Treated Neuro2a Cells

**Figure 2:**
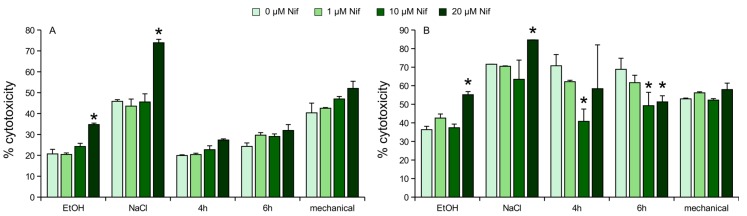
LDH activity measurement after stress on nifedipine pre-treated Neuro2a cells. (**A**) Undifferentiated Neuro2a; (**B**) differentiated Neuro2a. Values are given as the mean ± SD (error bars) of one representative out of at least three biologically independent experiments. Nif = nifedipine; mechanical = mechanical stress; NaCl = osmotic stress (150 mM); EtOH = oxidative stress (2%); 4 h = 4 h heat stress; 6 h = 6 h heat stress; single asterisk = *p* < 0.05 compared to non-treated cells.

In undifferentiated Neuro2a cells nifedipine was not able to reduce cytotoxicity induced by the investigated stressors, moreover it showed significantly higher cytotoxicity in higher doses, at least concerning osmotic and oxidative stress (Figure 2A). In detail, 20 µM nifedipine in combination with oxidative stress increases cell death from 25% to 34% (*p* < 0.005). Combined with osmotic stress, 20 µM nifedipine increases cell death from 46% to 74% (*p* < 0.005). None of the other treatments showed significant changes compared to corresponding non-treated controls.

In differentiated Neuro2a increase of cell death by combination of stress and nifedipine is also true for osmotic and oxidative stress. In heat-stressed cells, nifedipine was able to significantly reduce cell death in dosages of 10 and/or 20 µM compared to non-treated controls, but remains not as efficient as nimodipine (Figure 2B). In detail, 20 µM nifedipine in combination with oxidative stress increases cell death from 36% to 55% (*p* < 0.005). Combined with osmotic stress, 20 µM nifedipine increases cell death from 71% to 85% (*p* < 0.005). 10 µM nifedipine reduced cell death from 70% to 40% (*p* = 0.0045) when cells were incubated at 42 °C for 4 h. Concerning 6 h incubation at 42 °C, 10 and 20 µM reduced cell death from 68% to 49% (*p* < 0.05) and 51% (*p* < 0.05), respectively. None of the other treatments showed significant changes compared to corresponding non-treated controls.

### 2.3. Expression Levels of FA2H mRNA in Undifferentiated Neuro2a Cells

Nimodipine treatment enhances FA2H mRNA levels by 7–10 times compared to non-treated undifferentiated Neuro2a cells.

**Figure 3:**
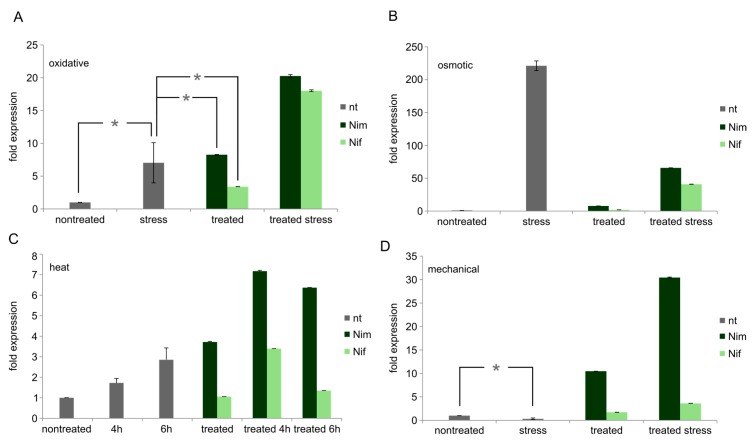
Fatty acid 2 hydroxylase (FA2H) mRNA levels in undifferentiated Neuro2a cells. Values are given as the mean ± SD (error bars) of one representative out of at least three biologically independent experiments. (**A**) Oxidative stress; (**B**) osmotic stress; (**C**) heat stress; (**D**) mechanical stress. nt = no drug pre-treatment; Nim = nimodipine pre-treatment (20 µM); Nif = nifedipine pre-treatment (20 µM); nontreated = no stress application; mechanical = mechanical stress; NaCl = osmotic stress (150 mM); EtOH = oxidative stress (2%); 4 h = 4 h heat stress; 6 h = 4 h; single asterisk = *p* > 0.05 (NOT significant) compared to each other. All other samples show *p* < 0.05 compared to each other each.

Oxidative (EtOH) stress increases FA2H expression to approx. 7× (*p* < 0.05). Nimodipine combined with EtOH leads to upregulation up to 20× (*p* < 0.005). Nifedipine alone increases FA2H mRNA level to approx. 3× (*p* < 0.005), but the combination of nifedipine and oxidative stress leads to an induction up to 18 times, which is statistically significantly lower than the induction achieved by nimodipine (*p* < 0.005) (Figure 3A).

Osmotic (NaCl) stress increases FA2H mRNA expression to approx. 220× (*p* < 0.005) and shows higher expression levels compared to nimodipine alone (*p* < 0.005). Also the combination of nimodipine and NaCl leads to an upregulation (65×, *p* < 0.005) of FA2H expression, but it remains lower than upregulation by NaCl alone. Nifedipine increases FA2H expression to 2× (*p* < 0.005). In combination with osmotic stress, nifedipine induces a 41× (*p* = 0.0001) higher FA2H expression. This also means, that nimodipine as well as nifedipine, the latter one even to a higher extend, repress the upregulation of FA2H induced by NaCl (Figure 3B).

Heat induces FA2H to approx. 2× (4 h, *p* < 0.005) or 3× (6 h, *p* ≤ 0.005), respectively. It is also upregulated (7× for 4 h, *p* < 0.005, and 6× for 6 h, *p* < 0.005) for heat-induced stress following nimodipine treatment and higher than in cells only treated with nimodipine. Nifedipine alone does not enhance FA2H mRNA levels, but in combination with 4 h heat stress an upregulation is detectable (3×, *p* < 0.005). This remains significantly lower than FA2H induction by nimodipine in corresponding conditions (*p* < 0.005) (Figure 3C).

Mechanical stress alone reduces FA2H expression approx. 3× (*p* < 0.005), and FA2H expression is higher (30×) for cells pre-treated with nimodipine and stressed by mechanical manipulation compared to reference cells, stressed cells or cells treated with nimodipine only (*p* < 0.005 each). Cells treated with nifedipine show also higher levels of FA2H mRNA, but they only reach an extension of 2× (nifedipine alone, *p* < 0.005) or 4× (nifedipine treatment followed by mechanical stress, *p* < 0.005) (Figure 3D). Asterisks in Figure 3 indicate NON-significance in comparison to relevant samples.

### 2.4. Expression Levels of FA2H in Differentiated Neuro2a Cells

Nimodipine treatment enhances FA2H mRNA levels up to 7–25 times compared to nontreated differentiated Neuro2a cells.

**Figure 4:**
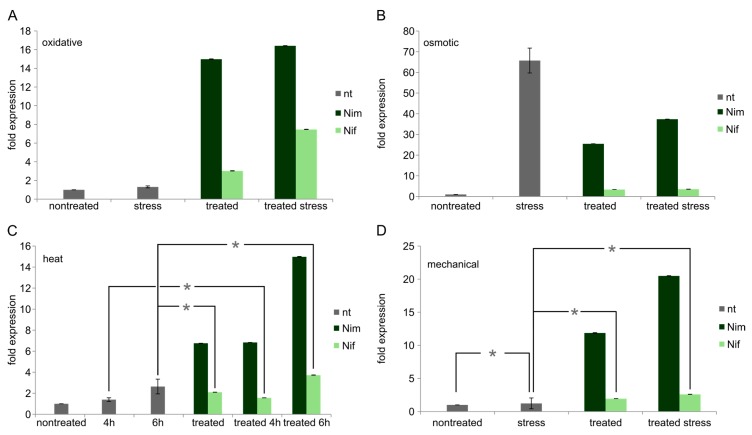
FA2H mRNA levels in differentiated Neuro2a cells. Values are given as the mean ± SD (error bars) of one representative out of at least three biologically independent experiments. (**A**) Oxidative stress; (**B**) osmotic stress; (**C**) heat stress; (**D**) mechanical stress. nt = no drug pre-treatment; Nim = nimodipine pre-treatment (20 µM); Nif = nifedipine pre-treatment (20 µM); nontreated = no stress application; mechanical = mechanical stress; NaCl = osmotic stress (150 mM); EtOH = oxidative stress (2%); 4 h = 4 h heat stress; 6 h = 6 h heat stress; single asterisk = *p* > 0.05 (NOT significant) compared to each other. All other samples show *p* < 0.05 compared to each other each.

Osmotic (NaCl) stress increases FA2H expression to approx. 66× (*p* < 0.005) and shows higher expression levels compared to nimodipine alone (25×, *p* < 0.005). Also the combination of nimodipine and NaCl leads to an upregulation (37×, *p* < 0.005) of FA2H expression, but it remains lower than upregulation by NaCl alone (*p* < 0.005). Nifedipine increases FA2H expression to 3.5x both with and without osmotic stress (*p* < 0.005 each). This also means, that nimodipine as well as nifedipine, the latter one even to an higher extend, repress the upregulation of FA2H induced by NaCl (*p* < 0.005) (Figure 4B).

Heat induces FA2H to approx. 1.4× (4 h, *p* < 0.05) and 3× (6 h, *p* < 0.05). It is also upregulated (7× for 4 h and 15× for 6 h, *p* < 0.005 each) for heat-induced stress following nimodipine treatment and at least slightly higher than in cells only treated with nimodipine. Nifedipine alone enhances FA2H mRNA levels (2×, *p* < 0.005) and also in combination with heat stress an upregulation is detectable (1.5× for 4 h and 4× for 6h, *p* < 0.005 each). This remains significantly lower than FA2H induction by nimodipine in corresponding conditions (*p* < 0.005 each) (Figure 4C).

Mechanical stress alone has no effect on FA2H expression, but FA2H expression is higher (20×) for cells pre-treated with nimodipine and subsequently stressed by mechanical manipulation compared to reference cells (*p* < 0.005), stressed cells (*p* < 0.005) or cells treated with nimodipine only (*p* < 0.005). Nimodipine alone increases FA2H expression 11× (*p* < 0.005). Cells treated with nifedipine show also higher levels of FA2H mRNA, but they only reach an extension of 2× (nifedipine alone, *p* < 0.005) or 2.5× (nifedipine treatment followed by mechanical stress, *p* < 0.005). This remains significantly lower than upregulation of FA2H by nimodipine in corresponding conditions (*p* < 0.005 each) (Figure 4D). Asterisks in Figure 4 indicate NON-significance in comparison to relevant samples.

Taken together, nimodipine significantly increases expression of FA2H in both undifferentiated and differentiated cells compared to reference cells and compared to cells treated with stressor alone, except for osmotic stress. In contrast to that the induction of FA2H by nifedipine remains considerably lower than the induction by nimodipine.

### 2.5. Protein Levels of FA2H in Undifferentiated Neuro2a Cells

**Figure 5:**
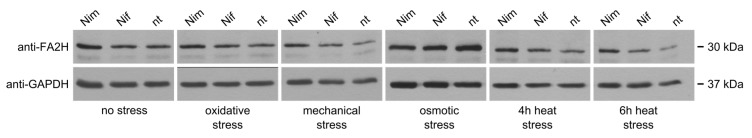
FA2H protein levels in undifferentiated Neuro2a cells. 20 µg total protein was loaded per sample on a 12% sodium dodecyl sulfate-polyacryl amide (SDS-PAA) gel. One representative out of at least three biologically independent experiments is displayed. Nim = nimodipine pre-treatment (20 µM); Nif = nifedipine pre-treatment (20 µM) nt = no drug pre-treatment; anti-FA2H = rabbit anti-FA2H antibody (1:1000); anti-GAPDH = mouse anti-GAPDH antibody (1:5000; loading control).

Nimodipine pre-treatment of undifferentiated Neuro2a cells leads to slightly higher levels of FA2H protein compared to non-treated or nifedipine pre-treated samples (Figure 5, no stress). Oxidative, mechanical and heat stress application to Neuro2a cells after nimodipine pre-treatment lead also to minor higher amounts of FA2H compared to nifedipine or non-treated cells (Figure 5, stress). FA2H protein is more strongly induced by osmotic stress alone than by nimodipine or nifedipine (Figure 5, osmotic stress).

### 2.6. Protein Levels of FA2H in Differentiated Neuro2a Cells

**Figure 6:**
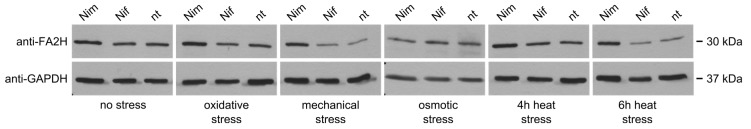
FA2H protein levels in undifferentiated Neuro2a cells. 20 µg total protein was on loaded per sample on a 12% SDS-PAA gel. One representative out of at least three biologically independent experiments is displayed. Nim = nimodipine pre-treatment (20 µM); Nif = nifedipine pre-treatment (20 µM) nt = no drug pre-treatment; anti-FA2H = rabbit anti-FA2H antibody (1:1000); anti-GAPDH = mouse anti-GAPDH antibody (1:5000; loading control).

Nimodipine pre-treatment leads to faintly higher levels of FA2H protein compared to non-treated or nifedipine-treated samples also in differentiated Neuro2a cells (Figure 6, no stress). Oxidative, mechanical and heat stress application to Neuro2a cells after nimodipine pre-treatment lead also to higher amounts of FA2H compared to nifedipine or non-treated cells (Figure 6, stress). Osmotic stress does not induce FA2H expression as strong as found in qPCR.

## 3. Discussion

Nimodipine is a dihydropyridine calcium antagonist with a long history and a good safety profile. Its neuroprotective effect has been shown in several clinical trials [5,11,12,13,16,26,27] and animal experiments [7,8,9]. We showed in 2014 that nimodipine is capable of protecting undifferentiated Neuro2a cells from cell death caused by mechanical, heat and oxidative stress. This was now proven for differentiated Neuro2a cells, which supports the idea of nimodipine acting neuroprotective.

In contrast to that, a protective effect of nifedipine concerning surgery-like stressors on Neuro2a cells was only observed concerning heat-induced stress on differentiated cells. This could be due to a special role of heat-shock proteins, which function as regulators for apoptotic cell death [28]. Also, Lecht et al. found in 2012 that nifedipine showed lower neuroprotective efficacy than nimodipine [19]. In other studies also nifedipine showed a beneficial effect on several cell lines [18,29,30], but no surgery-like stress models were included. Regarding the fact that nimodipine does not reduce cell death to very low levels but at least in part only slightly, we believe that the neuroprotective effect of nimodipine does not necessarily rely on its function as a Ca^2+^ blocking agent, but is rather a side effect. This could also be a reason that nifedipine does not show a neuroprotective impact and that no dose-dependency was observed. Furthermore, pre-conditioning that was done in this study is often known to be not dose-dependent.

However, very little is known about the molecular mechanism of the neuroprotection in general and for the mechanism traced back on nimodipine. Therefore we performed microarray analysis whereupon fatty acid 2-hydroxylase exhibited to be upregulated.

Sphingolipids play an important role in many types of molecular processes. 2-hydroxy fatty acid containing sphingolipids are common in nervous tissue and are components of myelin. About 1/3 of lipids found in myelin are galactosylceramides (GC) or sulfatides (3-sulfate esters of GC) in both, CNS and PNS [31,32,33]. Fatty acid 2-hydroxylase (FA2H) provides 2-hydroxy fatty acids for de novo synthesis of GC and sulfatides [23,34,35], although myelination can be performed without 2-hydroxy fatty acids at least in mice [33,36]. In this case, myelin is unstable and tends to degrade [33,37]. Therefore FA2H seems to be required for the production of 2-hydroxy GCs in order to provide stability of myelin sheath [33]. Mutations in the FA2H-gene lead to leukodystrophy with spastic paresis and dystonia [38]. Upregulation of fa2h may lead to a more actual myelination or re-myelination after nerve tissue damage during surgery. Wang et al. showed that nimodipine promotes re-myelination of peripheral nerves after injury [10]. Moreover, Zhang and David suggest that L-type Ca^2+^ channels, the target of nimodipine, are only active in paranodal demyelination, but not in intact myelinated axons [39]. This suggests that nimodipine in vivo can only work in myelin damaged nerves.

All these findings are in consistence with our findings of FA2H mRNA and protein upregulation by nimodipine. Nimodipine increases levels of FA2H mRNA as well as FA2H protein in both undifferentiated and differentiated cells to higher extent as seen in reference cells and stressed cells, except for osmotic stress. However, mRNA and proteins levels cannot be directly linked to each other. This may be due to translational regulation processes. The upregulation of FA2H may contribute to better survival of stressed Neuro2a cells pre-treated with nimodipine—also except for osmotic stress—shown previously by our workgroup [20]. Especially in mechanical stress, which is strongly linked to surgical manipulation in patients, regulation of FA2H without nimodipine may be analogous to processes in vivo. In none of the analyzed conditions an upregulation of FA2H by nifedipine was observed. The fact that nifedipine does not upregulate FA2H is in contrast to the assumption that the protective effect of nimodipine is linked to Ca^2+^ channels.

In cell culture as well as in clinical approaches pre-treatment with nimodipine seems to be required for better hearing preservation and/or nerve function after vestibular schwannoma surgery and better survival of cells, respectively [14,15,20]. While optimal pre-treatment period for cell culture is 24 h [20], best pre-treatment period for patients still needs to be defined.

Up to now we do not know any medication for protection of peripheral nerve tissue during surgery besides nimodipine. Unfortunately many patients suffer from hearing loss and/or loss of facial nerve function in vestibular schwannoma surgery, although nerve tissue itself is not damaged. We want to put more effort on the development of molecular processes in order to care for better life quality after surgery.

The potential function of nimodipine in processes of remyelination could open new possibilities in regeneration of myelin. Especially diseases linked to demyelination such as MS or injuries require remyelination [40]. At least in the CNS myelin sheaths can regenerate [41] and are targets of therapies in MS [42]. In addition to known strategies for remyelination therapy such as cell transplantations or antibody-based strategies [43], the induction of genes involved in remyelination could be an approach for neurodegenerative diseases.

Nimodipine, but not nifedipine, increases the expression of FA2H and therefore may lead to better (re-)myelination of nerves after surgery. We want to analyze this coherence in more detail in order to find better prophylactic treatment during neurosurgical interventions.

## 4. Materials and Methods

### 4.1. Value of Experiments

All tests in this study were at least performed as three biological replicates.

### 4.2. Cell Culture

Neuro2a cells are used to study neuronal differentiation, axonal growth, and signaling pathway [44].

Neuro2a cells were cultured in Dulbecco’s Modified Eagle Medium (DMEM, Thermo Fisher Scientific, Waltham, MA, USA) supplemented with 5% Fetal Calf Serum (FCS, Thermo Fisher Scientific) for non-differentiated cells and 2.5% FCS for differentiated cells, respectively, 1% Non-essential amino acids (NEAA, Thermo Fisher Scientific), and penicillin/streptomycin (100 U/mL/100 mg/mL, Thermo Fisher Scientific) in cell culture 150 cm^2^ plastic flasks (Greiner, Frickenhausen, Germany) under humidified atmosphere with 5% CO_2_ at 37 °C.

### 4.3. Nimodipine/Nifedipine Treatment

1 × 10^5^ cells were seeded on 24-well plates (Greiner) for LDH assays or 1 × 10^6^ cells were seeded on 100 mm-dishes (TPP, Trasadingen, Switzerland) for qPCR or Western blot, respectively, and incubated with 0, 1, 10, or 20 µM nimodipine or nifedipine (f.c.), respectively, diluted from a 1000× stock solution in EtOH abs. 24 h prior to stress application. Equal amounts of EtOH were added to non-treated controls.

### 4.4. Stress Application

Cell stress was induced and following groups were analyzed:

- Oxidative stress: Nimodipine or nifedipine pre-treated and control cells were treated with 2% EtOH.
- Osmotic stress: Nimodipine or nifedipine pre-treated and control cells were treated with 150 mM sodium chloride (NaCl).
- Heat: Nimodipine or nifedipine pre-treated and control cells have been incubated at 42 °C for 4 h or 6 h.
- Mechanical stress: Nimodipine or nifedipine pre-treated and control cells were shaken with two 2 mm steel beads at 500 rpm for 30 s. Afterwards steel beads were magnetically removed.

After stress treatment cells were incubated for another 24 h under humidified atmosphere with 5% CO_2_ at 37 °C.

All tests were performed as technical triplicates.

### 4.5. Lactate Dehydrogenase (LDH) Assay

Cytotoxicity was analyzed by using the Cytotoxicity Detection Kit (LDH, Roche, Grenzach-Wyhlen, Germany) following the manufacturer’s instructions. In brief, 100 µL freshly prepared reaction mixture were added to each 100 µL of the cell culture medium and incubated for 20 min. Absorbance was measured at 492 nm. Adding 2% Triton X-100 to the cells resulted in totally lysis and served as positive control (100% cell death). All tests were performed as biological triplicates, each of which as technical triplicate (three wells per sample per plate). Wells were measured 4 times.

### 4.6. RNA Isolation and cDNA Synthesis

Total RNA was isolated by RNeasyMaxiKit (Qiagen, Hilden, Germany) and eluted in 30 µL H_2_O. cDNA was then synthesized via RevertAid First Strand Synthesis Kit (Thermo Fisher Scientific) according to the manufacturer’s instructions using oligodT primer and 11 µL of the appropriate RNA.

### 4.7. qPCR

Quantitative PCR (qPCR) was carried out using SyGreen Mix (Nippon Genetics, Düren, Germany) on a StepOnePlus Cycler (Thermo Fisher Scientific). Threshold cycle (95 °C for 3 s and 60 °C for 60 s) for each PCR amplification was calculated by the cycler software. The specificity of PCR reaction was verified by melting curve analysis of the amplified product for each sample. The oligonucleotide sequences and their conditions for use are summarized in Table 1. GAPDH was used to normalize expression levels. Reference cells’ gene expression was always set to value 1, and fold expression refers to this. Fold expression was calculated using the ΔΔ*C*_t_-method.

All tests were performed as technical triplicates.

### 4.8. Protein Preparation

Cells were harvested using trypsinisation. Cell pellets were washed twice with PBS and afterwards resuspended in 50 µL RIPA buffer (Millipore Corp., Darmstadt, Germany) containing HALT protease and phosphatase inhibitor cocktail (Thermo Fisher Scientific). Samples were incubated on ice for 30 min while shaking to lyse the cells and subsequently centrifuged at 4 °C at 13,000 rpm for 20 min. Supernatants were transferred to a new tube.

### 4.9. Protein Assay

Total protein was determined by using BioRad Protein Assay (BioRad, Munich, Germany) according to the manufacturer’s instructions. In brief, 200 µL of diluted reagent was added to 10 µL of an appropriate dilution of total protein samples in a 96 well plate (TPP). After 10 min incubation, absorbance was measured at 595 nm using an InfinitePro 200 plate reader (Tecan, Männedorf, Switzerland). Protein concentrations were calculated according to a standard curve.

### 4.10. Western Blotting

Equal amounts of total proteins were separated using 12% SDS-PAGE and electrophoretically transferred to a PVDF membrane. Antibodies (rabbit anti-FA2H 1:1000 dilution, PA5-24728, Thermo Fisher Scientific; mouse anti-GAPDH 1:5000 dilution, MA5-15738, Thermo Fisher Scientific) were incubated with blots overnight at 4 °C, followed by incubation with secondary antibodies conjugated with horseradish peroxidase (Sigma-Aldrich, Munich, Germany) for 1 h at room temperature. The enhanced chemiluminescence (Pierce ECL, Thermo Fisher Scientific) was used to detect the bands.

### 4.11. Statistical Analysis

Significance was determined using one-way ANOVA as well as Student’s *t*-test. The reference for the *p*-values is the nimodipine non-treated sample. Statistical significant differences are presented at probability levels of *p* < 0.05 as results of both methods.

## 5. Conclusions

Nimodipine, but not nifedipine, increases the expression of FA2H and therefore may lead to better (re-)myelination of nerves after surgery.

## Figures and Tables

**Table 1:** Oligonucleotides used for qPCR. FA2H = oligonucleotide for *fa2h*; GAPDH = oligonucleotide for *gapdh*; fwd = forward; rev = reverse; Tm = melting temperature.

Oligonucleotide	Sequence (5′→3′)	T_m_ (°C)	Fragment Size
qFA2H_fwd	GGAGAAGTATGATGAGTGGGTTC	62	295 bp
qFA2H_rev	GAAGCGGTGGATGACGTATT	62	
qGAPDH_fwd	GGAGAAACCTGCCAAGTATGA	62	223 bp
qGAPDH_rev	CCTGTTGCTGTAGCCGTATT	62	

## References

[1] National Institute for Health and Clinical Excellence (NICE) Hypertension in Adults: Diagnosis and Management. Clinical Guideline CG127. https://www.nice.org.uk/guidance/cg127.

[2] De Lemons J.A., O’Rourke R.A., Fuster V. (2008). Unstable angina and non-ST-segment elevation myocardial infarction. Hurst’s the Heart.

[3] Thompson A.E., Pope J.E. (2005). Calcium channel blockers for primary Raynaud’s phenomenon: A meta-analysis. Rheumatology.

[4] King J.F., Flenady V.J., Papatsonis D.N., Dekker G.A., Carbonne B. (2003). Calcium channel blockers for inhibiting preterm labour. Cochrane Database Syst. Rev..

[5] Dorhout Mees S., Rinkel G.J., Feigin V.L., Algra A., van den Bergh W.M., Vermeulen M., van Gijn J. (2007). Calcium antagonists for aneurysmal subarachnoid haemorrhage. Cochrane Database Syst. Rev..

[6] Rabinstein A.A., Lanzino G., Wijdicks E.F. (2010). Multidisciplinary management and emerging therapeutic strategies in aneurysmal subarachnoid haemorrhage. Lancet Neurol..

[7] Hydman J., Remahl S., Björck G., Svensson M., Mattsson P. (2007). Nimodipine improves reinnervation and neuromuscular function after injury to the recurrent laryngeal nerve in the rat. Ann. Otol. Rhinol. Laryngol..

[8] Kansu L., Ozkarakas H., Efendi H., Okar I. (2011). Protective effects of pentoxifylline and nimodipine on acoustic trauma in Guinea pig cochlea. Otol. Neurotol..

[9] Nishimoto K., Kumai Y., Sanuki T., Minoda R., Yumoto E. (2013). The impact of nimodipine administration combined with nerve-muscle pedicle implantation on long-term denervated rat thyroarytenoid muscle. Laryngoscope.

[10] Tang Y.D., Zheng X.S., Ying T.T., Yuan Y., Li S.T. (2005). Nimodipine-mediated re-myelination after facial nerve crush injury in rats. J. Clin. Neurosci..

[11] Bischoff B., Romstöck J., Fahlbusch R., Buchfelder M., Strauss C. (2008). Intraoperative brainstem auditory evoked potential pattern and perioperative vasoactive treatment for hearing preservation in vestibular schwannoma surgery. J. Neurol. Neurosurg. Psychiatry.

[12] Roh J.L., Park C. (2008). A prospective, randomized trial for use of prednisolone in patients with facial nerve paralysis after parotidectomy. Am. J. Surg..

[13] Scheller C., Strauss C., Fahlbusch R., Romstöck J. (2004). Delayed facial nerve paresis following acoustic neuroma resection and postoperative vasoactive treatment. Zent. Neurochir..

[14] Scheller C., Richter H.P., Engelhardt M., Köenig R., Antoniadis G. (2007). The influence of prophylactic vasoactive treatment on cochlear and facial nerve functions after vestibular schwannoma surgery: A prospective and open-label randomized pilot study. Neurosurgery.

[15] Scheller C., Wienke A., Tatagiba M., Gharabaghi A., Ramina K.F., Ganslandt O., Bischoff B., Zenk J., Engelhorn T., Matthies C. (2016). Prophylactic nimodipine treatment for cochlear and facial nerve preservation after vestibular schwannoma surgery: A randomized multicenter Phase III trial. J. Neurosurg..

[16] Scheller K., Scheller C. (2012). Nimodipine promotes regeneration of peripheral facial nerve function after traumatic injury following maxillofacial surgery: An off label pilot-study. J. Craniomaxillofac. Surg..

[17] Nuglisch J., Karkoutly C., Mennel H.D., Roßberg C., Krieglstein J. (1990). Protective effect of nimodipine against ischemic neuronal damage in rat hippocampus without changing postischemic cerebral blood flow. J. Cereb. Blood Flow Metab..

[18] Daschil N., Humpel C. (2014). Nifedipine and nimodipine protect dopaminergic substantia nigra neurons against axotomy-induced cell death in rat vibrosections via modulating inflammatory responses. Brain Res..

[19] Lecht S., Rotfeld E., Arien-Zakay H., Tabakman R., Matzner H., Yaka R., Lelkes P.I., Lazarovici P. (2012). Neuroprotective effects of nimodipine and nifedipine in the NGF-differentiated PC12 cells exposed to oxygen-glucose deprivation or trophic withdrawal. Int. J. Dev. Neurosci..

[20] Herzfeld E., Strauss C., Simmermacher S., Bork K., Horstkorte R., Dehghani F., Scheller C. (2014). Investigation of the neuroprotective impact of nimodipine on Neuro2a cells by means of a surgery-like stress model. Int. J. Mol. Sci..

[21] Boggs J.M., Koshy K.M., Rangaraj G. (1988). Influence of structural modifications on the phase behavior of semi-synthetic cerebroside sulfate. Biochim. Biophys. Acta.

[22] Löfgren H., Pascher I. (1977). Molecular arrangements of sphingolipids. The monolayer behaviour of ceramides. Chem. Phys. Lipids.

[23] Maldonado E.N., Alderson N.L., Monje P.V., Wood P.M., Hama H. (2008). FA2H is responsible for the formation of 2-hydroxy galactolipids in peripheral nervous system myelin. J. Lipid Res..

[24] Pascher I. (1976). Molecular arrangements in sphingolipids. Conformation and hydrogen bonding of ceramide and their implication on membrane stability and permeability. Biochim. Biophys. Acta.

[25] Stewart R.J., Boggs J.M. (1993). A carbohydrate-carbohydrate interaction between galactosylceramide-containing liposomes and cerebroside sulfate-containing liposomes: Dependence on the glycolipid ceramide composition. Biochemistry.

[26] Ljunggren B., Brandt L., Säveland H., Nilsson P.E., Cronqvist S., Andersson K.E., Vinge E. (1984). Outcome in 60 consecutive patients treated with early aneurysm operation and intravenous nimodipine. J. Neurosurg..

[27] Sittel C., Sittel A., Guntinas-Lichius O., Eckel H.E., Stennert E. (2000). Bell’s palsy: A 10-year experience with antiphlogistic-rheologic infusion therapy. Am. J. Otol..

[28] Garrido C., Gurbuxani S., Ravagnan L., Kroemer G. (2011). Heat shock proteins: Endogenous modulators of apoptotic cell death. Biochem. Biophys. Res. Commun..

[29] Kasza Á., Hunya Á., Frank Z., Fülöp F., Török Z., Balogh G., Sántha M., Bálind Á., Bernáth S., Blundell K.L. (2016). Dihydropyridine Derivatives Modulate Heat Shock Responses and have a Neuroprotective Effect in a Transgenic Mouse Model of Alzheimer’s Disease. J. Alzheimers Dis..

[30] Pauwels P.J., Van Assouw H.P., Peeters L., Leysen J.E. (1990). Neurotoxic action of veratridine in rat brain neuronal cultures: Mechanism of neuroprotection by Ca^2+^ antagonists nonselective for slow Ca^2+^ channels. J. Pharmacol. Exp. Ther..

[31] Hoshi M., Williams M., Kishimoto Y. (1973). Characterization of brain cerebrosides at early stages of development in the rat. J. Neurochem..

[32] Kishimoto Y., Radin N.S. (1959). Isolation and determination methods for brain cerebrosides, hydroxy fatty acids, and unsaturated and saturated fatty acids. J. Lipid Res..

[33] Kota V., Hama H. (2014). 2′-Hydroxy ceramide in membrane homeostasis and cell signaling. Adv. Biol. Regul..

[34] Alderson N.L., Maldonado E.N., Kern M.J., Bhat N.R., Hama H. (2006). FA2H-dependent fatty acid 2-hydroxylation in postnatal mouse brain. J. Lipid Res..

[35] Eckhardt M., Yaghootfam A., Fewou S.N., Zöller I., Gieselmann V. (2005). A mammalian fatty acid hydroxylase responsible for the formation of alpha-hydroxylated galactosylceramide in myelin. Biochem. J..

[36] Potter K.A., Kern M.J., Fullbright G., Bielawski J., Scherer S.S., Yum S.W., Li J.J., Cheng H., Han X., Venkata J.K. (2011). Central nervous system dysfunction in a mouse model of FA2H deficiency. Glia.

[37] Zöller I., Meixner M., Hartmann D., Büssow H., Meyer R., Gieselmann V., Eckhardt M. (2008). Absence of 2-hydroxylated sphingolipids is compatible with normal neural development but causes late-onset axon and myelin sheath degeneration. J. Neurosci..

[38] Edvardson S., Hama H., Shaag A., Gomori J.M., Berger I., Soffer D., Korman S.H., Taustein I., Saada A., Elpeleg O. (2008). Mutations in the fatty acid 2-hydroxylase gene are associated with leukodystrophy with spastic paraparesis and dystonia. Am. J. Hum. Genet..

[39] Zhang Z., David G. (2016). Stimulation-induced Ca^2+^ influx at nodes of Ranvier in mouse peripheral motor axons. J. Physiol..

[40] McMurran C.E., Jones C.A., Fitzgerald D.C., Franklin R.J. (2016). CNS Remyelination and the Innate Immune System. Front. Cell Dev. Biol..

[41] Franklin R.J., Goldman S.A. (2015). Glia Disease and Repair-Remyelination. Cold Spring Harb. Perspect. Biol..

[42] Rodgers J.M., Robinson A.P., Miller S.D. (2013). Strategies for protecting oligodendrocytes and enhancing remyelination in multiple sclerosis. Discov. Med..

[43] Harlow D.E., Honce J.M., Miravalle A.A. (2015). Remyelination Therapy in Multiple Sclerosis. Front. Neurol..

[44] Tremblay R.G., Sikorska M., Sandhu J.K., Lanthier P., Ribecco-Lutkiewicz M., Bani-Yaghoub M. (2010). Differentiation of mouse Neuro 2A cells into dopamine neurons. J. Neurosci. Methods.

